# Urban Bat box translocation for *Vespertilio sinensis* conservation

**DOI:** 10.1038/s41598-025-08661-4

**Published:** 2025-07-02

**Authors:** Yanze Zhou, Guanjun Lu

**Affiliations:** School of Geographical Sciences, Changchun Normal University, Changchun, Jilin China

**Keywords:** Bat, Bat box, Urban habitat, Bat activity levels, Conservation, Animal behaviour, Biogeography

## Abstract

Bats are vital for ecosystem stability, yet their natural habitats are increasingly threatened by urbanization, resulting in marked population declines. While some species have adapted to artificial structures as roosting sites, public fear and prejudice often lead to bat displacement, exacerbating human-bat conflicts. Therefore, exploring feasible solutions to increase bat roosting habitats in urban areas is urgent. On July 25, 2019, 40 individuals of *Vespertilio sinensis* (20 adults and 20 subadults) were artificially translocated from residential areas in Changchun, Jilin Province, to a new building using bat box. Over the 114-day observation period, the population dynamics of *V. sinensis* roosting in the bat box exhibited five distinct phases, characterized by a gradual decline in total bat numbers. In the year of installation, activity levels of *Pipistrellus abramus* and *Hypsugo alaschanicus* substantially decreased but returned to pre-installation levels the following year. Continuous monitoring from 2020 to 2022 indicated that although the activity levels of *V. sinensis* increased, not many *V. sinensis* individuals continued to use the bat box. The results of this study provide foundational data for the establishment of artificial bat roosts in urban areas and offer scientific insights for mitigating human-bat conflicts and promoting harmonious urban ecosystem development.

**Supplementary Information:**

The online version contains supplementary material available at 10.1038/s41598-025-08661-4.

## Introduction

Bats play a crucial role in ecosystem services as seed dispersers, pollinators, insect controllers, and nutrient recyclers, making them essential indicators of ecosystem health^1–3^. However, public knowledge about bats remains limited. Bats are often linked to zoonotic diseases^4,5^, a perception partially supported by their role as natural reservoirs for RNA viruses^6^. Nevertheless, the actual prevalence of these diseases within bat populations has been substantially exaggerated^7^. Another common misconception is that all bats exhibit hematophagy. However, existing studies indicate that only three bat species are valid sanguivorous: *Desmodus rotundus*, *Diaemus youngi*, and *Diphylla ecaudata*, all of which are distributed in the Americas^8^. These misconceptions foster negative biases and even fear towards bats^9^.

Urbanization, the dominant driver of land-use alteration, fundamentally alters the landscape and ecological functions within and beyond urban boundaries, substantially modifying wildlife habitats^10^. Dense forests, caves, and old trees, which traditionally provide suitable habitats for bats are increasingly being replaced by high-rise buildings and extensive road networks. This transformation poses severe challenges, particularly for bat species that rely strictly on caves or tree hollows for roosting^11^. Meanwhile, many bat species have demonstrated remarkable adaptability to anthropogenic structures. For instance, *Tadarida teniotis* has learned to utilize crevices in high-rise walls as cliff-like roosting sites, while *Rhinolophus ferrumequinum* often selects spacious attic areas as maternity colonies^12^. However, due to prevailing fears, misconceptions, as well as issues such as the odor and noise caused by bats, homeowners often take measures to drive bats away^13^. Given the escalating human-bat conflicts in urban environments, exploring feasible strategies to enhance bat habitat availability in cities outside private owners’ houses has become a critical component of bat conservation efforts.

Bat boxes have emerged as a highly promising conservation tool for preserving bat species diversity, offering suitable alternative habitats for bat populations displaced by human activities and direct eviction^14^. Substantial progress has been achieved in utilizing bat boxes as substitute roosts, with a variety of designs developed, including rectangular wooden boxes, wood-cement composite boxes (constructed from a blend of sawdust and cement), and multi-chambered boxes. The combination of architectural features and material composition in these designs generates diverse localized climate conditions^15^. The occupancy rate of bat boxes is influenced by multiple factors. Given that bats prefer communal roosting, multiple bat boxes should be installed within a specific area, with a recommended density ranging from 2 to 8 boxes per 10 hectares. The ideal placement locations should take into account various considerations, such as the distance of bat boxes from water sources and their orientation towards sunlight based on species-specific preferences, in order to enhance the occupancy rate of bat boxes^16^. Additionally, non-target species, such as birds and non-flying mammals, may occupy bat boxes, thereby reducing the number of available boxes for bats^17^. The reproductive status of bats can also affect their use of bat boxes^18^. However, long-term evaluations regarding the utilization of bat boxes by artificially translocated bat populations in temperate urban ecosystems remain scarce.

*Vespertilio sinensis* is a medium-sized insectivorous bat distributed across multiple regions, including China, Korea, and Japan. This species originally primarily roosted in tree hollows and rock crevices. However, with the rapid development of urbanization, *V. sinensis* has increasingly adapted to utilizing artificial structures as roosting sites^19^. This study aims to investigate the feasibility and effectiveness of artificially translocating *V. sinensis* from residential areas to a new urban habitat by using a single bat box as the roosting site. Specifically, we seek to achieve the following objectives: (1) evaluate the occupancy and utilization of the bat box by *V. sinensis* after translocation to the new habitat; (2) assess the potential impact of newly introduced *V. sinensis* on the activity levels of pre-existing bat species in the target habitat; (3) monitor whether *V. sinensis* will continue to use the bat box for roosting over the following years.

We predict that there will be a short-term peak in bats leaving the bat box initially after translocation, followed by a gradual and stable establishment of roosting within the bat box. The translocation process may temporarily disrupt the local existing bat population, but a stable coexistence relationship is expected to form over time. Moreover, it is anticipated that most of the translocated individuals will continue to use the newly installed bat box over the following years. The findings of this study will provide crucial baseline data and scientific insights for urban bat conservation efforts.

## Results

### Changes in the emergence number and activity level of *V. sinensis*

**Fig. 1:**
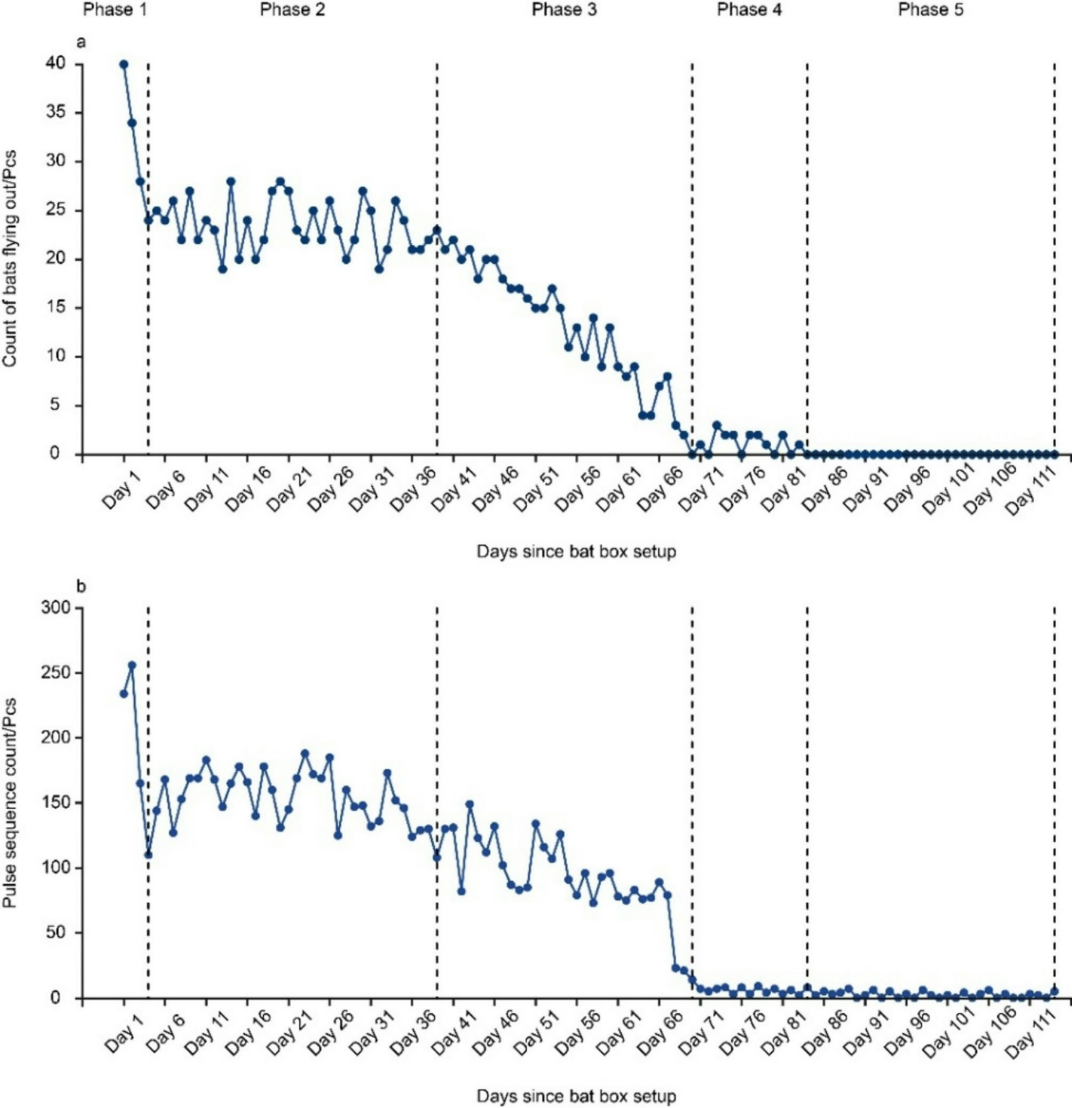
Temporal changes in the emergence counts of *V. sinensis* flying out of the bat box (**a**) and the echolocation pulse sequence counts of *V. sinensis* (**b**).

**Fig. 2:**
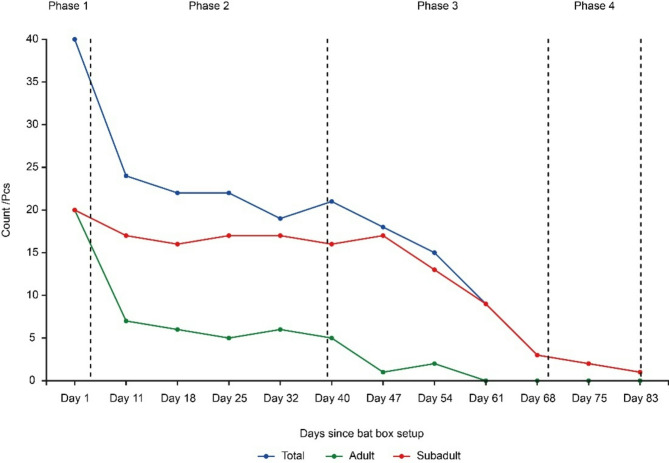
The temporal changes in adult, subadult, and total *V.sinensis* counts in the bat box (only node data).

On the day of the bat box installation, a total of 40 *V. sinensis* individuals were placed, consisting of 20 adults and 20 subadults. The number of *V. sinensis* individuals emerging from the bat box throughout 2019 was divided into five phases:

- Phase 1 (Day 1–Day 4, July 25–July 28): A sharp decline in *V. sinensis* emergence was observed (Fig. 1a). Observations of the individual composition in the bat box indicated that this decrease was primarily due to the reduction in the number of adult *V. sinensis*, whereas the subadult population remained relatively stable (Fig. 2).
- Phase 2 (Day 5–Day 39, July 29–September 2): The number of *V. sinensis* flights fluctuated but exhibited an overall stable trend (Fig. 1a). No substantial changes were observed in the number of either adults or subadults (Fig. 2).
- Phase 3 (Day 40–Day 70, September 3–October 2): During this phase, the number of *V. sinensis* flights declined steadily and continuously (Fig. 1a), primarily due to the concurrent decrease in both adult and subadult populations (Fig. 2). By the end of this phase, all adult *V. sinensis* had departed.
- Phase 4 (Day 71–Day 83, October 3–October 15): The number of subadult *V. sinensis* continued to decline (Fig. 2).
- Phase 5 (Day 84–Day 114, October 16–November 15): No *V. sinensis* flights were recorded (Fig. 1a).

The trend in activity levels of *V. sinensis* mirrored the pattern of emergence counts (Fig. 1b). However, following the disappearance of individuals from the bat box in Phase 5, a minimal level of bat activity was still observed.

### Impact of Bat box installation on the activity levels of foraging Bats

**Fig. 3:**
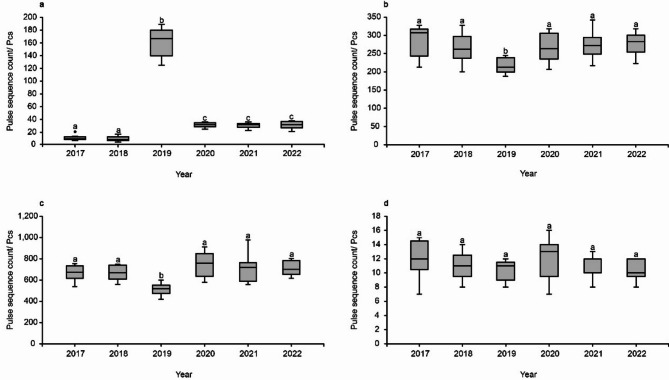
Differences in (**a**) *V. sinensis*, (**b**) *P. abramus*, (**c**) *H. alaschanicus*, and (**d**) *M. ikonnikovi* activity levels every August from 2017 to 2022 (data come from 9 sessions each year).

Every August from 2020 to 2022, significant differences were observed in the activity levels of *V. sinensis* (*P* = 0.0000000204). The activity levels of *V. sinensis* during the three years following bat box installation were substantially higher than those in the two years preceding installation but substantially lower than in the year of bat box installation (Fig. 3a). Significant inter-annual differences in activity levels were also noted for *Pipistrellus abramus* (*P* = 0.008213) and *Hypsugo alaschanicus* (*P* = 0.00000403). In the year of bat box installation, the activity levels of *P. abramus* and *H. alaschanicus* were substantially lower than those observed in both the two years prior to and the three years following the installation. However, no substantial differences were found between the two years prior and the three years post-installation for either species (Fig. 3b and c). No significant differences were found in the activity levels of *Myotis ikonnikovi* (*P* = 0.4275, Fig. 3d).

From August 2020 to August 2022, *V. sinensis* was observed flying out of the bat box on 11 out of 27 observation nights each year. Specifically, there were 3 nights in 2020, 5 in 2021, and 3 in 2022, with only one individual observed per night. On 16 occasions, no bats were observed roosting in the bat box. Although the activity level of *V. sinensis* increased significantly from 2020 to 2022, few *V. sinensis* continued to roost in this bat box.

## Discussion

A study investigating social dynamics in *Rhinopoma microphyllum* demonstrated the effects of intraspecific competition on foraging efficiency. The study found that when inter-individual distances fell below 12 m, bats allocated more attention to conspecific avoidance. The consistent presence of conspecifics within the bats’ sensory range resulted in a notable decrease in their predatory attacks on prey during a brief period^20^. Therefore, the limited space within an abandoned factory is insufficient to support the foraging activities of both the original *V. sinensis* population and the newly introduced *V. sinensis* bats in the bat box without interference. This interference effect likely contributed to the mass departure of adult *V. sinensis* during Phase 1. In phase 2, the number of bats in the box substantially decreased, reducing the interference effects, which consequently led to a stabilization in the number of adult bats. There may be suitable habitats for bats in the areas surrounding the factory. Although we have not conducted a detailed survey of potential bat habitats in the vicinity, we can hypothesize that the departure of the first batch of adult bats might also be due to their translocation to these surrounding habitats.

During August and September in Ontario, Canada, there was no substantial difference in food intake between adult and subadult *Myotis lucifugus*. However, despite similar food intake, adult *M. lucifugus* exhibited a trend of weight increase, while subadult bats exhibited weight loss. This phenomenon suggests that subadults exhibited a negative energy balance (energy expenditure > intake)^21^. This could be due to the relatively lower foraging efficiency of subadult bats, which require higher foraging costs to achieve the same energy intake as adults^22^. Prior to the installation of the bat box, this abandoned factory served as a habitat for four bat species, and foraging behaviors of these four bat species were also recorded at this location. The proximity of the bat box allowed subadult *V. sinensis* to forage nearby, thereby reducing their foraging costs and enabling them to accumulate the necessary fat reserves for hibernation^23^.

This factor may explain why the number of subadult *V. sinensis* in the bat box remained relatively stable during phases 1 and 2.

*V. sinensis* primarily preys on Lepidoptera, and the population of Lepidoptera is substantially influenced by temperature factors^24,25^. In the third phase of the abandoned factory, the temperature exhibited a decreasing trend compared to the second phase. This temperature change may have caused a shift in the structure of food resources available in the abandoned factory, prompting large numbers of *V. sinensis* to leave the area. Bats in temperate regions, due to food scarcity and low temperatures in winter, need to undertake seasonal migration or seek hibernacula with suitable temperature and humidity^26^. Although no research explicitly identifies the exact hibernation period of *V. sinensis*, reference to *R. ferrumequinum*, which is also found in Jilin Province, reveals that its hibernation period can last 6 to 8 months^27^. Therefore, it can be inferred that the departure of both adult and subadult *V. sinensis* from the bat box in phase 3 might also be related to seasonal migration or the search for more suitable hibernacula.

A study investigated the hibernation characteristics of juvenile *M. daubentonii* and *M. nattereri* (individuals born in the same year) in North Rhine-Westphalia, Germany. The study found that these juvenile individuals hibernated for a substantially shorter period than adults, with later onset and earlier arousal from hibernation^28^. This may be due to the relatively low-fat reserves in juvenile individuals, which are insufficient to support long periods of hibernation. Additionally, early arousal helps them forage earlier, thereby increasing their survival rate^29,30^. This finding explains why, after all the adult *V. sinensis* had left the bat box in phase 3, subadult *V. sinensis* continued to roost in the bat box until phase 5. Bats wake up during hibernation to drink and forage. Therefore, the absence of *V. sinensis* in the bat box during phase 5, while still observing the activity of *V. sinensis* in the abandoned factory, may indicate that a few bats that were hibernating nearby woke up to drink or forage^31,32^.

There is an overlap in foraging and roosting areas between *Pipistrellus sp.* and *M. mystacinus* in Poland, leading to a substantial negative correlation between their populations. Specifically, in areas with a higher population of *Pipistrellus sp.*, the population of *M. mystacinus* was relatively lower^33^. Given that all four bat species were engaged in foraging activities within this abandoned factory (predatory buzzes were recorded), the artificial translocation initiative caused a sharp rise in the number of *V. sinensis* foraging nearby at night in the area. This change could potentially have an impact on *P. abramus* and *H. alaschanicus*, which originally foraged in this region at night. However, since not many *V. sinensis* continued to use the bat boxes in the factory for roosting, this may have led to a decrease in the level of interspecific competition in the area from 2020 to 2022. As a result, over the three years following the installation of the bat box, the activity levels of *P. abramus* and *H. alaschanicus* returned to their pre-installation levels. Additionally, the activity levels of *M. ikonnikovi* did not show significant variations each August from 2017 to 2022. This might be because the foraging range of this species within the abandoned factory was relatively limited, thereby shielding it from the notable impact of the surge in *V. sinensis* population.

During the three years following the installation of the bat box, the activity levels of *V. sinensis* in the abandoned factory significantly increased compared to the two years prior. This might be because, from 2020 to 2022, some of the relocated *V. sinensis* continued to be active within the abandoned factory but did not continue to use the bat box for roosting. Different bat species exhibit varying preferences for the shape and volume of bat boxes, and conspecific bats also use different bat boxes for mating, rearing, and hibernation depending on the season^16^. *V. sinensis* also shows different preferences in bat box use; those with prior breeding experience tend to prefer bat boxes in shaded environments, while bats without breeding experience prefer bat boxes exposed to sunlight. The number of *V. sinensis* using the bat box during the lactation period was low, and all of them were non-reproductive. However, after the lactation period ended, the number of *V. sinensis* using the bat box substantially increased, including both those with breeding experience and those without^18^. The design of the bat box used in this experiment was random and did not take into account the habitat preferences of *V. sinensis*, which may be the primary reason why, in the three years following the translocation, not many *V. sinensis* continued to use the bat box in the factory.

## Conclusion

Following the artificial translocation of *V. sinensis* to a novel habitat, its utilization of the bat box exhibited phased variations, primarily driven by age-dependent factors and seasonal fluctuations. Due to the urgency of finding suitable wild habitats for the captured bats to ensure their protection, this experiment had certain limitations. For instance, we did not mark or track the captured *V. sinensis* individuals. Nor did we investigate the original population size of *V. sinensis* in the new habitat. Consequently, we were unable to observe the numerical comparison between the original and newly translocated *V. sinensis* populations after translocation. Additionally, a detailed survey of potential bat habitats surrounding the abandoned factory was not conducted.

In this experiment, the newly introduced *V. sinensis* individuals increased interspecific competition within the new habitat, leading to a decline in the activity levels of two original foraging species in the same year. However, their activity levels recovered to pre-translocation levels within three years of the bat box‘s establishment. Although only a small number of *V. sinensis* continued to use the bat box over the three-year period, their activity levels significantly increased compared to those before the establishment of the bat box. We consider these to be positive outcomes, which suggest that the artificial translocation of *V. sinensis* did not have a negative impact on the original bat species in the long run and instead enhanced the activity levels of *V. sinensis* in the habitat. This provides valuable references for future in-depth studies on the artificial translocation of bats using bat boxes, while also highlighting the importance of long-term observations in bat translocation experiments, rather than solely focusing on the year of translocation.

In future experiments involving the translocation of bat species using bat boxes, it is crucial to comprehensively consider the size of the new habitat and the number of bats to be translocated to avoid foraging interference. Meanwhile, different bat species, as well as the same species in different seasons and reproductive stages, exhibit distinct preferences for bat boxes. Future bat box designs should account for species-specific preferences.

## Methods

### Sample source

A large number of *V. sinensis* roosted on the fifth-floor balcony of a residential building located in the 34th Street area of the Economic Development Zone in Changchun, Jilin Province (https://www.sohu.com/a/327468826_115464). This caused severe disturbances to nearby residents, including noise pollution and occasional intrusions into their living spaces, preventing them from opening windows for ventilation at night. Despite efforts by the Changchun Fire Rescue Brigade to remove the bats, they continued to return and roost in the gaps of the balcony, further disrupting the daily lives of the local population.

### Sample transfer

From July 17 to July 21, 2019, a total of 40 *V. sinensis* were captured from the balcony of the residential building. These included 20 adult females, 11 female subadults, and 9 male subadults (a few weeks old). Each bat was individually placed in a clean white cotton bag. With the consent of the residents, the bats were transferred to the Animal Behavior and Conservation Laboratory at Changchun Normal University. On the first day of the transfer, fecal samples from the subadult bats were collected, softened with glycerin, and then evenly spread on microscope slides. By analyzing the diet composition using a biological microscope, it was confirmed that they were capable of independent predation^34,35^. During the day, the captured bats were temporarily housed in a bat cage (65 cm × 35 cm × 35 cm). Half an hour after sunset each day, they were fed mealworms and an appropriate amount of purified water. After feeding, the bats were placed in a white mosquito net (3 m × 2 m × 2 m) to fly freely.

### Sample placement site selection and placement

The finally selected site for the artificial translocation was an abandoned factory located near the Changji North Line in the Erdao District of Changchun (coordinates: 43.92°N, 125.40°E; area: 332.94 m × 162.78 m). The factory was selected for the following reasons: (1) it was abandoned and uninhabited, eliminating the potential for disturbance to residents (no residential zones within 400 m); (2) there was no artificial lighting or noise disturbance in the factory at night; (3) In August 2017 and 2018, we monitored the nocturnal activity of bats in the factory every 3–4 days, conducting a total of 9 monitoring sessions each year. The monitoring was carried out using the same instruments, instrument placement positions, and monitoring methods as those employed in the 2019 experiment. Preliminary monitoring data indicated that four bat species were active in the factory at night, providing baseline data for evaluating the impact of *V. sinensis* migration on other bat species. Therefore, based on these factors, the location was deemed suitable for the artificial translocation of *V. sinensis*. On July 25, 2019, we placed bats into the bat box and hung it 2.5 m above the ground in a room within the abandoned factory. The main body dimensions of the bat box are 27 cm × 18 cm × 12 cm. An opening is provided at the bottom of the box for bats to enter and exit. The basic materials used are all 1 cm-thick beech wood planks in their natural color. To prevent the impact of odors on bats, the materials are placed in an open space with direct sunlight and good air circulation for drying before fabrication. The room had a door and a window on the south side and a window on the north side. In this study, bat capture and captive management procedures were conducted in strict compliance with applicable Chinese laws and regulations. The involved bat species are not listed as nationally or regionally protected. Throughout the research process, we strictly adhered to animal welfare principles, ensuring minimal interference with and impact on bat populations. All experimental protocols received approval from the Science and Technology Ethics Committee of Changchun Normal University. The study is reported in accordance with ARRIVE guidelines.

### *V. sinensis* bat box use and factory bat activity monitoring

From each evening between July 25 and November 15, 2019, we employed two methods to determine the number of *V. sinensis* in the bat box and to assess the dynamic changes in bat activity levels at the study site: (1) Direct census of bat emergence from the bat box. Thirty minutes before sunset, we conducted observations near the bat box to count the number of exiting *V. sinensis*. At this time, there was sufficient ambient light to allow for clear counting without compromising visibility (a single observer maintained continuous observation for 1.5 h). (2) Nocturnal acoustic sampling. On the day of monitoring, acoustic recording devices were placed in an open, fixed location 8 m horizontally from the bat box (only one monitoring device was used). From 30 min before sunset to 30 min after sunrise the following day, we recorded the echolocation calls of bats. We used Kaleidoscope Pro analysis software (version 5.1.9, https://www.wildlifeacoustics.com/products/kaleidoscope-pro) to quantify the activity level of each species by counting the number of echolocation pulse sequences (where each sequence consisted of one or more echolocation pulses, and consecutive pulses with intervals of less than 1 s were considered as a complete pulse sequence). Starting from the 11th day after the installation of the equipment, we observed the individual composition within the bat box every 7 to 8 days, recording the numbers of adult and subadult bats. Furthermore, in the subsequent years, we conducted nine monitoring sessions each August at the study site to assess bat activity levels (with monitoring carried out every 3 to 4 days). Simultaneously, we observed the emergence numbers of *V. sinensis* from the bat boxes (using the same experimental methodology as in 2019).

### Data statistics and analysis

The activity levels of *V. sinensis*, *P. abramus*, *H. alaschanicus*, and *M. ikonnikovi* every August from 2017 to 2022 (*n =* 54) were tested for normality and homogeneity of variance using Shapiro-Wilk and Levene tests. The activity levels of *V. sinensis* and *P. abramus* in 2017 violated the assumption of normality. Moreover, annual activity levels (2017–2022) of the four species were normally distributed each year, but the August activity levels of all species except *P. abramus* did not exhibit homogeneity of variance. Thus, the Kruskal-Wallis test was applied to the August activity levels of *V. sinensis* and *P. abramus* over six years. If significant differences were found (*P* < 0.05), Dunn’s multiple comparisons were performed. For *H. alaschanicus* and *M. ikonnikovi*, Welch ANOVA was applied to their August activity levels over six years, and post-hoc comparisons using the Games-Howell test were conducted if significant differences were found (*P* < 0.05). These analyses quantified the differences in August activity levels among the four bat species over the six years. All multiple comparisons were corrected using the Bonferroni method, and all P-values reported are Bonferroni-adjusted P-values. All statistical analyses were performed using R v4.4.0, and all plotting was carried out using Origin and Adobe Illustrator.

## Electronic supplementary material

Below is the link to the electronic supplementary material.

Supplementary Material 1

## Data Availability

The data that support the findings of this study are available from the corresponding author upon reasonable request.
