# Supplementary material for: Urban Bat box translocation for Vespertilio sinensis conservation
Source: Sci Rep. 2025 Jul 2;15:23473. doi: 10.1038/s41598-025-08661-4 (PMC12223148; doi:10.1038/s41598-025-08661-4)
Supplement: Supplementary file 1 — Supplementary Material 1 [file 41598_2025_8661_MOESM1_ESM.docx]

1. normal distribution test

totalee_data <- read.csv("D:/total3/totalb.csv")

shapiro_test_result <- shapiro.test(totalee_data$C)

print(shapiro_test_result)

2. homogeneity of variance test

totalee_data <- read.csv("D:/total3/totalb.csv")

head(totalee_data)

library(tidyr)

long_data <- gather(totalee_data, key = "group", value = "value", A:F)

long_data$group <- factor(long_data$group)

head(long_data)

# install.packages("car")

library(car)

levene_result <- leveneTest(value ~ group, data = long_data)

print(levene_result)

3. Kruskal-Wallis test and Dunn's multiple comparisons (default bonferroni)

totalee_data <- read.csv("D:/total3/totalb.csv")

head(totalee_data)

library(tidyr)

library(dunn.test)

long_data <- gather(totalee_data, key = "group", value = "value", A:F)

head(long_data)

kruskal_result <- kruskal.test(value ~ group, data = long_data)

print(kruskal_result)

if (kruskal_result$p.value < 0.05) {

dunn_result <- dunn.test(long_data$value, long_data$group, kw = TRUE, label = TRUE)

print(dunn_result)

}

4. welch_anova test and games_howell_test (bonferroni)

library(rstatix)

library(ggplot2)

data <- read.csv("D:/total3/totald.csv")

head(data)

data_long <- reshape(data,

varying = c("A", "B", "C", "D", "E", "F"),

v.names = "value",

timevar = "group",

times = c("A", "B", "C", "D", "E", "F"),

direction = "long")

head(data_long)

data_long$group <- as.factor(data_long$group)

welch_anova <- oneway.test(value ~ group, data = data_long, var.equal = FALSE)

print(welch_anova)

if (welch_anova$p.value < 0.05) {

gh_results <- data_long %>%

games_howell_test(value ~ group) %>%

adjust_pvalue(method = "bonferroni")

print(gh_results)

}

totala=*Vespertilio sinensis*

totalb= *Pipistrellus abramus*

totalc= *Hypsugo alaschanicus*

totald= *Myotis ikonnikovi*
